# Editorial for Special Issue “Multifunctional Nanomaterials and Hybrid Structures for Sensors, Actuators and Smart Technologies”

**DOI:** 10.3390/nano13040722

**Published:** 2023-02-14

**Authors:** Sharmila M. Mukhopadhyay, Mallikarjuna Nadagouda

**Affiliations:** 1 Frontier Institute for Research in Sensor Technologies (FIRST), The University of Maine, Orono, ME 04469, USA; 2 Graduate School, Wright State University, Dayton, OH 45435, USA

Advanced materials related to sensing, actuation, catalysis, and other functionalities for interactive devices depend on surface interactions and quantum effects in solids. These phenomena are significantly enhanced in nanomaterials, which provide an ultra-high surface area per unit mass/volume, and ample scope for bandgap engineering. These materials can help address many current humanitarian challenges, ranging from clean water, renewable energy, and eco-friendly infrastructures to disaster relief, public health, and national security. These opportunities have motivated this Special Issue, entitled “Multifunctional Nanomaterials and Hybrid Structures for Sensors, Actuators and Smart Technologies”, which has attracted research papers related to a variety of emerging nanomaterials, as outlined below.

Organic complexes such as porphyrins can be useful for multiple applications. In particular, a mixed substituted A3B porphyrin and its Pt complex were investigated [1] for various applications such as drug detection (hydroquinone), corrosion inhibition, and protective coating for water-splitting electrodes.

The latter application, protective coatings on devices, is becoming important for sustainable smart technologies that need to be deployed in challenging environments. One of the papers is focused on an investigation of novel passivation systems suitable for aggressive acid conditions [2]. Laser-assisted techniques were used to create smooth layers of mixed oxides and porphyrin-based films with low porosity that are extremely adherent to the surface of steel. Testing these as corrosion-resistant coatings for electrochemical electrodes revealed efficient and promising passivation, even in strong acids.

Silica-based hybrid nanomaterials are being recognized for potential catalytic applications related to environmental mitigation technologies. In particular, silica matrices containing platinum, either as nanoparticles or organometallic conjugates, can be very useful for the removal of dye from wastewater, and one of the studies [3] provides a detailed quantification of their capacity and efficiency for the removal of fuchsine B dyes.

Sensors to detect chemicals such as nitric oxide (NO) would be desirable technologies for the healthcare industry. Hybrids of single-walled carbon nanotubes (SWNTs) with specific DNA chains can be useful as fluorescence sensors for NO detection. A recently developed sensor platform was analyzed mathematically [4] to establish an easy method for NO quantification. These studies set a base for future sensor development.

Gold nanocrystals (Au NCs) have risen to the forefront of sensing, photovoltaic, and diagnostic research due to their unique shape and size-specific properties such as photoluminescence (PL), quantized double-layer charging, and electrochemiluminescence (ECL). The key to utilizing these properties for successful applications will depend upon how precisely their surfaces can be functionalized. One of the papers [5] investigates the post-synthesis functionalization of Au NCs for the enhancement of their photoluminescent and electro-chemo-luminescent properties.

Among nanomaterials for biochemical detection, graphene oxide (GO), with its excellent optical properties and biocompatibility, has attracted significant attention. A graphene oxide (GO)-coated microfiber was investigated as an ultrasensitive sensor for the in situ detection of hemoglobin [6]. This was shown to provide high sensitivity, low cost, small size, and fast response, hence offering a competitive alternative in the diagnosis of blood diseases.

Finally, advanced nanocatalysts are known to provide unprecedented performance and precision in a variety of applications related to sensing, chemical conversion, and environmental sustainability. However, the practical deployment of such catalysts would require supporting them on other matrices rather than deploying them as isolated nanoparticles. Nanocatalyst–support interactions are expected to impact the functionality of such materials, and investigation of the hybrid catalyst–support system becomes important. In particular, nano-palladium catalysts have shown significant promise in a large number of applications related to the degradation of aqueous contaminants, where they are supported on organic and inorganic matrices. An overview of recent investigations on matrix-supported Pd-nanocatalysts is presented in this volume [7], with some of the novel concepts highlighted as future technology platforms.

Overall, this volume provides a collection of selected papers covering different aspects of nanomaterials, and we hope the readers find this useful.
